# The gap between policy and practice: a systematic review of patient-centred care interventions in chronic heart failure

**DOI:** 10.1007/s10741-015-9508-5

**Published:** 2015-10-05

**Authors:** P. M. Kane, F. E. M. Murtagh, K. Ryan, N. G. Mahon, B. McAdam, R. McQuillan, C. Ellis-Smith, C. Tracey, C. Howley, C. Raleigh, G. O’Gara, I. J. Higginson, B. A. Daveson

**Affiliations:** King’s College London, Cicely Saunders Institute, Department of Palliative Care, Policy and Rehabilitation, Bessemer Road, London, SE5 9PJ UK; St. Francis Hospice, Dublin, Ireland; Mater Misericordiae University Hospital, Dublin, Ireland; Beaumont Hospital, Dublin, Ireland

**Keywords:** Patient-centred care, Heart failure, Systematic review, Palliative care, Shared decision-making

## Abstract

Patient-centred care (PCC) is recommended in policy documents for chronic heart failure (CHF) service provision, yet it lacks an agreed definition. A systematic review was conducted to identify PCC interventions in CHF and to describe the PCC domains and outcomes. Medline, Embase, CINAHL, PsycINFO, ASSIA, the Cochrane database, clinicaltrials.gov, key journals and citations were searched for original studies on patients with CHF staged II–IV using the New York Heart Association (NYHA) classification. Included interventions actively supported patients to play informed, active roles in decision-making about their goals of care. Search terms included ‘patient-centred care’, ‘quality of life’ and ‘shared decision making’. Of 13,944 screened citations, 15 articles regarding 10 studies were included involving 2540 CHF patients. Three studies were randomised controlled trials, and seven were non-randomised studies. PCC interventions focused on collaborative goal setting between patients and healthcare professionals regarding immediate clinical choices and future care. Core domains included healthcare professional-patient collaboration, identification of patient preferences, patient-identified goals and patient motivation. While the strength of evidence is poor, PCC has been shown to reduce symptom burden, improve health-related quality of life, reduce readmission rates and enhance patient engagement for patients with CHF. There is a small but growing body of evidence, which demonstrates the benefits of a PCC approach to care for CHF patients. Research is needed to identify the key components of effective PCC interventions before being able to deliver on policy recommendations.

**Electronic supplementary material:**

The online version of this article (doi:10.1007/s10741-015-9508-5) contains supplementary material, which is available to authorized users.

## Introduction

Chronic heart failure (CHF) is a life-limiting progressive condition [1, 2] predominantly affecting elderly patients with multiple co-morbidities [3]. Treatment advances have increased prognosis and treatment options with more patients now living with advanced CHF [4]. In a condition with a comparable mortality rate to cancer [5], patients experience a considerable illness burden [6], reduced quality of life [7] and high levels of uncertainty particularly for the future [8]. As treatment options have increased, treatment decisions have become more challenging for patients and clinicians [9]. This is compounded by patients who poorly understand their prognosis [10], overestimate the benefits of life-prolonging treatments [11] and fail to appreciate the detrimental effect these treatments can have on their quality of life [9]. Older patients may have a preference for prolonged independence, better cognitive and physical function over life-prolonging treatments, if given the informed opportunity to choose [12–14]. Patient-centred care (PCC) answers to this challenge by incorporating patients’ preferences, values, beliefs, illness understanding, illness experience and information needs into the decision-making process, thus encouraging patient engagement and collaborative goal setting [15, 16].

The National Institute for Health and Clinical Excellence (NICE) [17], the European Society of Cardiology [18] and the American Heart Association [9] have recommended a patient-centred approach for CHF. Health policy recommends a patient-centred approach [19–21], but an agreed global definition is lacking [22, 23]. Domains common to the concept of PCC in the literature include: respect for patients’ needs [19, 24–30], values [19, 25–27, 29–32] and preferences [19, 23–30, 32, 33], patient–healthcare professional collaboration [19, 22, 24–33] and shared decision-making [19, 23–28, 31–33]. In chronic illness—such as CHF—patients must navigate through complex information and treatment choices while experiencing the ramifications of chronic ill health on their lives. Health policy supports the role of patients as informed, active and prepared decision-makers in their own health care, rather than passive recipients [23, 29, 34–36]. In the move away from a paternalistic disease-focused approach, PCC actively encourages patient involvement [26] while recognising the patient as a ‘whole person’ rather than merely experiencing a disease process. In chronic illness, PCC has a beneficial effect on healthcare professional–patient concordance regarding treatment plans, patient health outcomes and patient satisfaction [37] and respects patients’ desired level of involvement in healthcare decisions [38, 39]. The central domains of PCC are also found in the concept of the palliative care approach to CHF management which explicitly views these PCC domains in the context of CHF as a life-threatening disease. Additionally, the palliative care approach states that its central goal is improvement of quality of life for both patient and family [40]. Fundamental to both is shared decision-making (SDM). Good PCC which is being examined here manifests as SDM; patient–healthcare professional collaboration ensures that patients’ values, needs and preferences are met and evidence and clinical experience guide the decision-making process [23, 28, 37, 39].

To our knowledge, no systematic review has examined the evidence for PCC interventions in CHF. This review therefore aims (i) to identify PCC interventions in CHF where patients’ are involved as informed, active participants in SDM about their clinical care and identify their own personal care goals and (ii) to describe domains of PCC included in the interventions and to describe the selected outcomes of these studies.

## Methods

With no agreed definition and heterogeneity in its operationalisation, assessing PCC as an effective approach to care presents a challenge. SDM, where healthcare professionals and patients are involved in making care decisions, involves a process of sharing information, identifying preferences and goals to reach common ground to enable the delivery of optimal health care to the patient [28, 30, 41, 42]. SDM has been identified as an essential component of PCC for CHF [9, 41]. It has been used in other systematic reviews as a reasonable indicator of PCC [42, 43]. As PCC implementation in clinical practice is a relatively new research area, a broad search strategy with a high sensitivity was preferred to a very specific search. A protocol was written, and a combination of database searches used in previous systematic reviews for PCC [42, 43], SDM [44] and quality of life [45] were modified based on scoping searches to include ‘patient empowerment’ and ‘self-care’ to increase sensitivity to intervention studies focusing on these PCC components. End-of-life care and advance care planning terms did not notably increase sensitivity and were omitted. Final search terms included ‘heart failure’ AND (‘patient-centred care’, OR ‘shared decision making’ OR ‘self-care’ OR ‘patient empowerment’) AND (‘quality of life’ OR ‘communication’ OR symptoms). Medline, Embase, PsycINFO, CINAHL, ProQuest ASSIA, Cochrane databases and clinicaltrials.gov were searched from inception to March 2015. This was supplemented by contacting authors, hand-searching bibliographies of PCC interventions reviews [8], key journals (European Journal of Heart Failure, Journal of Cardiac Failure) and citation and reference searches. ProQuest Dissertations and Theses Database were searched to capture unpublished literature (for search strategy, see Appendix of ESM).

One author (PMK) reviewed the abstracts and retrieved papers that fulfilled the criteria for closer scrutiny (Table 1). Two authors (PMK and CES) screened 10 % of abstracts to ensure agreement. Studies were included for data extraction if >40 % of participants had CHF (NYHA II–IV), the intervention included SDM and patient-centred outcome(s) were measured. Mixed studies were included where quantitative data fulfilled the inclusion criteria. Data extracted by PMK included: study design, intervention, setting, attrition rate, outcome(s) and PCC domains within interventions. Two authors (PMK and CES) assessed the quality of included studies using the Down and Black checklist for RCTs and non-RCTs [46]. Qualitative data were analysed using thematic analysis to identify PCC benefits or barriers [47]. Quantitative studies were to be analysed using pooled odds ratio or meta-analysis, if possible [48]. If not possible due to the number or type of studies or heterogeneity, results were to be analysed using the clustered intervention approach (with clusters consisting of interventions, outcomes or elements) and/or in tabular format to aid interpretation [49].

**Table 1:** Study inclusion criteria

Published studies were considered if they met the following eligibility criteria:
i) Adult population ≥18 years with chronic heart failure staged II–IV using the New York Heart Association (NYHA) classification, including both reduced ejection fraction and preserved ejection fraction
ii) In studies with mixed samples, at least 40 % have chronic heart failure and this population is reported on separately
iii) Studies can be of any quantitative or mixed-method design, except reviews or case studies/series
iv) An intervention will be included if the description of the intervention is adequate to allow the reviewer to establish that it aimed to increase patient-centred care behaviour by incorporating shared decision-making where this involved one or more of:
a. Promoting patient participation/involvement in the formulation of care plans
b. Shared control of the patient–healthcare professional consultation
c. Patient self-identification of their own goals of care
v) The intervention involved at least one face-to-face clinical consultation between the patient and healthcare professional
vi) Studies measured at least one health-related outcome, e.g. health-related quality of life (HRQoL), symptoms
vii) Studies in English

## Results

**Fig. 1:**
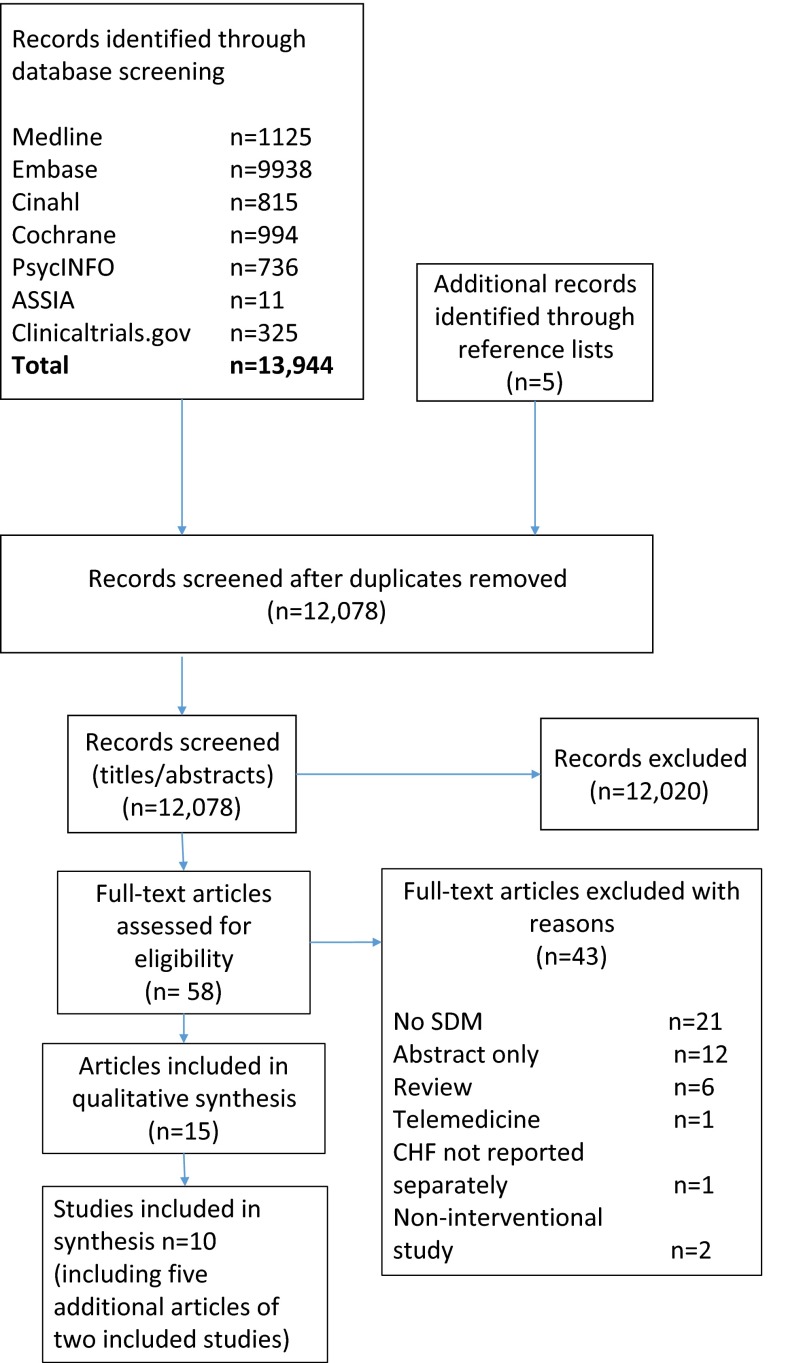
PRISMA flow diagram of study selection. *SDM* shared decision-making, *HCP* healthcare professional

The search retrieved 13,944 papers and a reference scan yielded 5 additional papers, as shown in the PRISMA flow diagram (Fig. 1) [50]. Of 12,078 papers screened at title and abstract, 12,020 papers were excluded, leaving 58 papers for full-text review. Forty-three papers were excluded as they did not fulfil the inclusion criteria. Fifteen papers were included regarding 10 studies with 3 additional articles regarding 1 study [51–53] and 2 additional articles regarding another study [54, 55].

A total of 2540 patients were included in 10 studies. Study characteristics are outlined in Table 2. 2 studies were based on an inpatient hospital setting [56, 57] with the remainder in outpatients or community settings. 3 studies used a mixed-method approach to explore patients’ perceptions of the PCC intervention [52, 53, 56, 58, 59]. Two explored perceived intervention acceptability and impact [57, 60].

**Table 2:** Characteristics of included studies (primary outcome and related results in bold)

First author, year, country	Design	Intervention	Participants, setting, diagnosis and attrition rate	Outcomes	Results	Quality assessment
Brannstrom, 2014, Sweden [60]	RCT, open, non-blinded 2 group parallel design, block randomisation, 1 − *β* = 0.80, *α* < 0.05, estimated attrition of 15 %, sample size estimate 31 patients needed in each arm	PREFER: Integrated heart failure and palliative care; medical assessment, then meetings/telephone nurse consultation Duration: 6 months Delivered by: multidisciplinary team including specialists in heart failure and palliative care, physiotherapy and occupational therapy	*n* = 72, Attrition rate 14 % Community setting Primary diagnosis of chronic heart failure 100 % had chronic heart failure, NYHA III–IV	**25** **% improvement in Mean Symptom burden compared with CG (ESAS: EDS)** HRQoL (EQ-5D) QoL (KCCQ) Functional class (NYHA) Hospitalisation Mortality Baseline, 1 m, 3 m, 6 m	**No significant difference in mean symptom burden (EDS) between IG and CG** **ESAS Nausea significantly improved in IG (p** **=** **0.02)** EQ-5D HRQoL increase at 6 m (8 % *p* = 0.013) (within group analysis in IG) KCCQ improvement in total symptom burden (18 %, *p* = 0.035), QoL (24 %, *p* = 0.047), self-efficacy (17 %, *p* = 0.041) (within group analysis in IG) NYHA improved in IG vs. CG (39 % 11 out of 28 vs. 7 %, 3 out of 32 *p* = 0.015) Fewer hospitalisations in IG vs CG (15 vs. 53, *p* = 0.009) Increased resource utilisation: nurse visits in IG vs. CG (1075 vs. 230, *p* = 0.0001) No difference in mortality	R:11 EV:3 IV (Bias):5 IV (Con):4 P:1 **Total:24**
Dionne-Odom, 2014, USA [58]	Before and after study, no control group, Phase II feasibility study	ENABLE: Palliative care consultation followed by weekly telephone delivered nurse coaching to patient following a prescribed format Patient and caregiver educational material Duration: 6 weeks (patient) 3 weeks (caregiver) Delivered by: Advance Practice Palliative Care Nurse	*n* = 22 comprising 11 patient/caregiver dyads, Attrition rate 55 % *n* = 15 clinicians Community setting, Primary diagnosis of chronic heart failure 100 % had chronic heart failure, NYHA III–IV	Patient depression and anxiety (HADS) Symptoms (MSAS-HF) HRQoL (KCCQ) Patient engagement (PACIC) Caregiver QoL (QOLC) Caregiver Burden (MBCB) Caregiver depression and anxiety (HADS) Baseline, 3 m, 6 m	Intervention feasibility study so descriptive statistics provided only with no significance levels. Follow-up numbers very small at 6 m (*n* = 5) Mean standardised effect size from baseline to week 24 showed improvements in patient HADS anxiety (−0.16), PACIC summary score (−0.08), Caregiver HADS Depression (−0.06), MBCB Stress Burden (−0.96)	R:7 EV:1 IV (Bias):5 IV (Con):3 P:0 **Total:16**
Shively, 2013, USA [61]	RCT, unblinded randomised 2 group repeated measures design, Phase III efficacy trial, 1 − *β* = 0.80, no sample size estimate or significance level available	Enhance self-management tailored to each patient’s level of activation Patient given ‘self-management toolkit’: DVD, educational booklet, pedometer, bp cuff, weight scale. 6 patients—nurse meeting to discuss patient’s individual goals Duration: 6 months Delivered by: Advance Practice Nurses	*n* = 84, Attrition rate 19 % Single-site outpatient study Primary diagnosis of chronic heart failure 100 % had chronic heart failure, NYHA I–III	**Patient activation (PAM: PAM Total Score)** Self-management (SCHFI, MOS Specific Adherence Scale) Hospitalisations ED attendances Baseline, 3 m, 6 m	**Increase in patient activation in IG vs CG, [71.5 (SD 17.43) vs. 64.4, (SD 15.40), p** **=** **0.03]** **Increase in patient’s perceived control, p** **=** **0.015 (PAM)** SCHFI no significant change MOS Specific Adherence Scale Improvement over time (*p* < 0.001) No significant reduction in hospitalisations or ED attendances	R:10 EV:2 IV (Bias):4 IV (Con):4 P:0 **Total:20**
Ekman, 2012, Sweden [50–52, 55]	Controlled before and after study, proof-of-concept study, 1 − *β* = 0.80, *α* < 0.05, Sample size estimate 91 patients in each group	PCC Intervention: 3-h education programme on theory and application of PCC to ward staff Duration: duration of patient admission Delivered by: ward staff	*n* = 248, Attrition rate 20.2 % Five medical wards in one hospital Primary diagnosis of chronic heart failure 100 % had chronic heart failure, NYHA I–IV	**LOS (length of hospital stay)** Activities of daily living (Katz ADL) HRQoL (KCCQ) Uncertainty (Ambiguity and complexity) (CPS) Hospital readmission Baseline, 3 m (ADL Baseline, discharge)	**Reduction in LOS by 1 day in IG vs. CG (9.22d vs. 8.22d, p** **=** **0.16), reduction in LOS by 2.5d in per protocol IG** ^**†**^ **(6.77d, p** **=** **0.01)** ADL improvement in functional capacity in IG vs. CG, *p* = 0.07 KCCQ No change in HRQoL CPS Reduction in uncertainty: Reduction in ambiguity, *p* = 0.041* Reduction in complexity, *p* = 0.02*, *p* = 0.024^†^ No difference in time to first readmission	R:10 EV:3 IV (Bias):5 IV (Con):4 P:1 **Total:23**
Evangelista, 2012, USA [64] [53], 2014 [54]	Prospective case–control study	Palliative care consultation Duration: 50–120 min Delivered by: Palliative Care Specialist or Advance Practice Palliative Care Nurse	*n* = 36, Attrition rate 14.3 % Single-centre outpatients Primary diagnosis of chronic heart failure 100 % had chronic heart failure, NYHA II–III	**Symptom burden (ESAS: EDS)** HRQoL (MLHFQ); Depression (PHQ-9) Patient-perceived control (CAS-R) Patient activation (PAM) Advance Directive Attitude Survey (ADAS) Baseline, 3 m	**ESAS Reduction in mean symptom burden (EDS), p** **=** **0.031** MLHFQ Improvement in overall score *p* < 0.035 PHQ-9 Improvement in depression *p* < 0.034 PAM Increased patient activation, *p* < 0.001 ADAS Increase in completion of advance directives *p* = 0.016	R:11 EV:2 IV (Bias):4 IV (Con):3 P:0 **Total:20**
Schellinger, 2011, USA [65]	Prospective cohort study	Respecting choices Disease Specific Advance Care Planning (DS-ACP) Video and discussion guide for ACP providers 26-h competency-based communication skills training programme Provision of disease-specific planning tools	*n* = 1894, Attrition rate 67.2 % Multi-site involving primary, inpatient and homecare Primary or secondary diagnosis of chronic heart failure	*Process measures* Referral number and source; Patient uptake of DS-ACP *Outcome measures* Documentation of resuscitation guidelines, Advance directives, Statement of treatment preferences; Hospice use and length of stay of deceased participants; Emergency department (ED) attendance or inpatient admissions within 30/60 days of referral	31.8 % (602) of referred patients completed DS-ACP, HCP referral significant association with DS-ACP participation (*p* < 0.001) No difference in resuscitation documentation Statement of treatment of preferences (84.8 % of DS-ACP participants vs. 0 % non-participants, *p* < 0.001) Health directive (94.3 % of DS-ACP participants vs. 24.8 % of non-participants, *p* < 0.001) DS-ACP participants twice as likely to participate in hospice compared non-participants (*p* = 0.003) 56.1 % of participants enrolled in hospice vs. 37.2 % of non-participants (*n* = 286 deceased participants), Mean LOS differed by 27.5d (DS-ACP participants vs. non-participants, 71.4d vs. 43.8d) No difference in readmissions or ED attendances between two groups	R:9 EV:3 IV (Bias):3 IV (Con):3 P:0 **Total:18**
Schwarz, 2012, USA [56]	Retrospective non-randomised cohort study	Palliative care consultation Delivered by Specialist Palliative Care Team	*n* = 20, attrition rate 0 % Single-site tertiary care setting 100 % had chronic heart failure, NYHA IV	Indication for palliative care referral Impact of palliative care consultation on patient care Completion of advance care directives (ACD)	Indications for palliative care referral in descending order (*n* = number of patients): symptom management (20); clarification of goals of therapy (7); advance care planning (6); hospice referral (2); end-of-life care (4). Moderate impact of palliative care referral on patient care 6 (30 %) patients completed ACD	R:5 EV:2 IV (Bias):3 IV (Con):2 P:0 **Total:12**
Delaney, 2010, USA [59]	Controlled before and after study, Feasibility study	Nurse-delivered education, Patient assessment at each of 8 visits, Nurse and patient education materials Therapeutic activities Duration: 8 weeks Delivered by: Cardiac Nurses	*n* = 24, Attrition rate 0 % Homecare agency Primary diagnosis of heart failure 100 % had chronic heart failure, NYHA III–IV	QoL (MLHFQ) Depression (PHQ-9) HEART Post-intervention survey (included 3 open-ended questions on acceptability of intervention) Hospital admission rates Baseline, 90d	MLHFQ Improved quality of life in IG vs. CG (*p* = 0.007) PHQ-9 Mean overall improvement in depressive symptoms in IG vs. CG, (3.0 vs. 1.1, *p* = 0.001) Lower hospital readmission rates in IG vs. CG (16 % vs. 25 %, non-significant)	R:9 EV:2 IV (Bias):2 IV (Con):3 P:0 **Total:16**
Riegel, 2006, USA [57]	Before and after study, no control (mixed methods)	Home visits (3–4) using a motivational approach Nurse trained in motivational approach and family counselling Duration: 3 months Delivered by: Advance Practice Nurse	*n* = 24, Attrition rate 38 % Community setting, Primary diagnosis of heart failure 100 % had chronic heart failure, NYHA II–IV	Self-care (SCHFI) Knowledge about heart failure (Representations questionnaire) Baseline, 6 m	SCHFI Improvement in patient self-care in 80 %, no significance level given	R:6 EV:0 IV (Bias):3 IV (Con):2 P:0 **Total:11**
Shively, 2005, USA [62]	RCT, non-blinded, 2 group parallel design, 1 − *β* = 0.80, *α* < 0.05, sample size estimate 34 in intervention group, 37 in control group	Behavioural management programme using an information–behaviour–motivation model (2 classes and 4 telephone calls) Emphasis on patient individualised goal setting Duration: 15 weeks Delivered by: nurse	*n* = 116, Attrition rate 13 % Single-site outpatients Primary diagnosis of heart failure 100 % had chronic heart failure, NYHA I–III	**Exercise Performance (6** **min walk test)** HRQoL (MLHFQ) Physical and mental functioning, general health perceptions (SF-36V) Functional ability (SAS) Baseline, 4, 10, 16 m	**6-min walk test: no significant difference in exercise** **performance** No difference in physical functioning, mental functioning or general health perceptions Improvement in physical functioning in MLHFQ physical dimension score, *p* = 0.03	R:10 EV:2 IV (Bias):4 IV (Con):5 P:1 **Total:22**

Primary outcomes are identified in bold

* Unadjusted per protocol population, ^†^ adjusted per protocol (PP) population (when patient-centred care was fully implemented)

PCC: Patient-centred care; CAS-R: Control Attitude Scale; PAM: Patient Activation Measure; ESAS: Edmonton Symptom Assessment System; EDS: ESAS Distress Score; IG: Intervention Group; CG: Control Group; HRQoL: Health-Related Quality of Life; MLHFQ: Minnesota Living with Heart Failure Questionnaire; PHQ-9: Patient-Health Questionnaire-9; EQ-5D: Euro QoL-5D; QoL: Quality of Life; KCCQ: Kansas Cardiomyopathy Questionnaire; ADL: Activities of Daily living; PAM: Patient Activation Measure; SCHFI: Self-Care of Heart Failure Index; MOS: Medical Outcomes Study Specific Adherence Scale; SF-36V: Medial Outcomes Study Short-Form Health Survey, Veterans adapted version; SAS: Specific Activity Scale; CAS-R: Control Attitude Scale-revised; HADS: Hospital Anxiety and Depression Scale; MBCB: Montgomery Borgalia Caregiver Burden Scales; QOLC: Caregiver quality of life; PACIC: Patient Assessment of Care for Chronic Conditions; MSAS: Memorial Symptom Assessment Scale; CPS: Cardiovascular Population Scale; ED: Emergency Department; Quality Appraisal Tool: Domains are R:reporting; EV: External Validity; IV (Bias): Internal Validity—bias; IV (Cons): Internal validity—confounding (selection bias); P: Power; HCP: Healthcare Professional; SD; Standard deviation

Sample size ranged from 24 to 1894, with an average age of 75 years and a high attrition rate. Three studies were phase II RCTs [61–64]. Two non-RCTs were controlled before and after studies [56, 60]. A meta-analysis was not possible due to the small number and heterogeneity of included studies. The majority of participants were male, NYHA functional class II–III with at least 3 co-morbidities. While all interventions involved SDM (defined earlier), the tools and techniques used were heterogeneous. The median quality score was 20 (possible total score of 32) (Table 2). The majority of papers scored well on reporting (median 10.5, possible total of 11) and external validity (median 3, possible total of 3) with poorer scores on internal validity (median 7, possible total of 13, combined score for selection and confounding bias) and power (median 0.0 possible total of 5).

A framework of commonly identified PCC domains was compiled from a literature review [19, 22–33]. Table 3 shows this framework and lists the common PCC domains together with the patterns of emphasis in included studies. The study by Ekman et al. [56] which involved PCC implementation at ward level and the studies which involving specialist palliative care as an intervention [57, 59, 61, 65] included most patient-centred domains. In addition to SDM, patient–healthcare professional collaboration, patient involvement in identification of goals of care, ascertainment of patient’s treatment preferences and patient activation were the most commonly identified domains.

**Table 3:** Table of patient-centred care domains

Patient-centred care domains	First author, year									
	Brannstrom, 2014 [59]	Dionne-Odom, 2014 [57]	Shively, 2013 [60]	Ekman, 2012 [54]	Schwarz, 2012 [55]	Evangelista, 2012 [52, 62], 2014 [53]	Delaney, 2010 [58]	Schellinger, 2011 [63]	Riegel, 2006 [56]	Shively, 2005 [61]
Physical, psychosocial assessment	✓	✓		✓	✓	✓	✓			
Patient activation	✓		✓	✓		✓	✓	✓	✓	
Patient motivation						✓			✓	
Patient involved in identification of goals of care	✓	✓	✓		✓	✓		✓	✓	✓
Shared decision-making	✓	✓	✓	✓	✓	✓	✓	✓	✓	✓
Ascertainment of patient’s treatment preferences	✓	✓		✓	✓	✓	✓	✓		
Barriers and problem-solving		✓	✓	✓		✓	✓		✓	
Family/caregiver involvement		✓			✓	✓		✓		✓
Patient–healthcare professional collaboration	✓	✓	✓	✓	✓	✓	✓		✓	✓
Patient and family/caregiver support	✓	✓		✓		✓			✓	✓
Patient partnership	✓			✓						
Care coordination		✓			✓	✓				

**Fig. 2:**
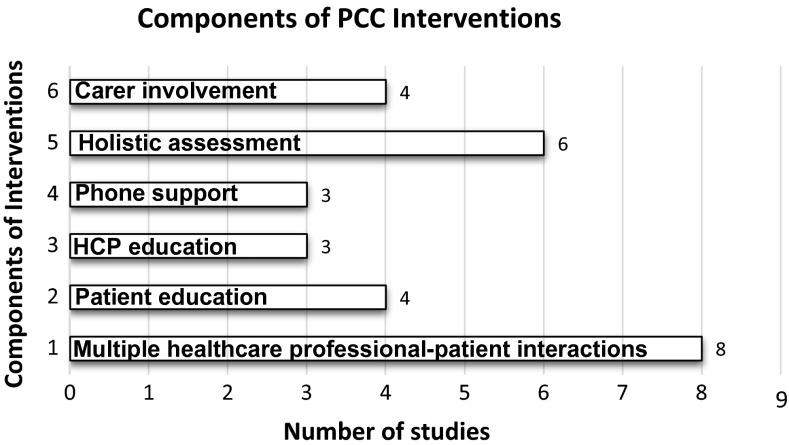
Separate components of PCC interventions across included studies

The common components of the interventions are shown in Fig. 2.

### Holistic assessment

Six studies included comprehensive assessments of patients’ physical, psychosocial [56, 60, 66] and spiritual needs [59, 61, 65] which provided information on patients’ understanding of their illness, its impact on their lives and their care preferences.

### SDM

Decision content ranged from immediate healthcare choices to advance care planning. Five studies involved advance care planning [57, 59, 61, 65, 66]. Specialist palliative care initiated and was involved in these discussions in 4 of these studies [57, 59, 61, 65]. In the implementation study by Schellinger et al. [66], trained facilitators discussed advance care planning with patients. Five studies focused on more immediate symptom management [56, 58, 60, 62, 63], of which 3 used motivational techniques to achieve greater concordance between patients’ goals and values and their current behaviour [58, 62, 63].

### Education and training

Seven studies included an educational component [56, 58–60, 62, 63, 66], of which 3 involved healthcare professional education and training [56, 58, 66]. Ekman et al. [56] provided a 3-h introduction on the theory and application of PCC to ward staff. In the Riegel et al. [58] study, a nurse was trained in a motivational approach and family counselling prior to providing patient home visits. Schellinger et al. [66] implemented the Respecting Choices Disease Specific Advance Care Planning (DS-ACP) [67] where trained facilitators received 26 h of competency-based communication skills training. Delaney et al. [60] provided a manual on guidelines to nurses delivering the intervention and an patient education booklet. Shively et al. [63] provided a patient education booklet with a nurse-delivered behavioural management programme. Dionne-Odom et al. [59] gave patients a workbook which they completed with nursing support. Shively et al. [62] gave patients an educational booklet and DVD.

### Multidisciplinary approach

Brannstrom et al. [61] was the only study to use a multidisciplinary approach to deliver PCC. Patients were given access to specialists (nurses and physicians) in palliative care and CHF care, together with physiotherapists and occupational therapists.

### Support

Family support was investigated in six interventions [56, 57, 61, 63, 65]. One study found that a lack of family support could act as a barrier to accessing available care [58].

## Outcome measures

**Fig. 3:**
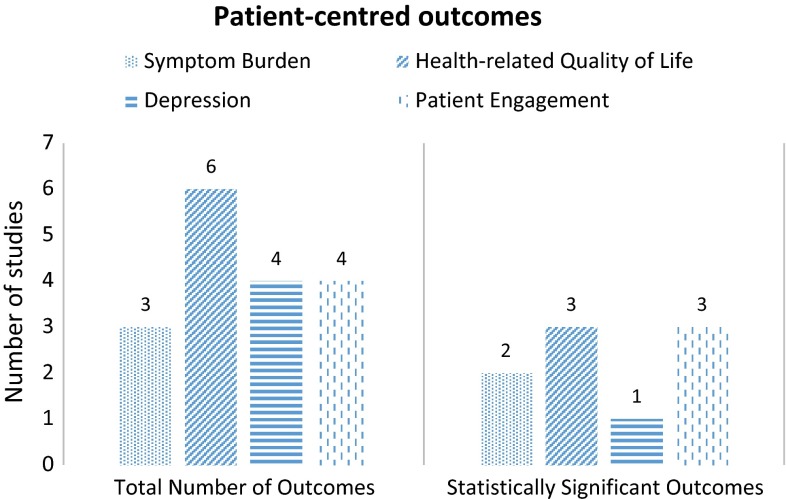
Total number of patient-centred outcomes in included studies and statistically significant patient-centred outcomes

The outcomes are outlined in Table 2 and in Fig. 3.

### Health-related quality of life (HRQoL)

Six studies measured HRQoL using the Kansas City Cardiomyopathy Questionnaire (KCCQ) [56, 59, 61] or the Minnesota Living with Heart Failure Questionnaire (MLHFQ) [60, 63, 65]. Delaney et al. [60], Evangelista et al. [65] and Brannstrom et al. [61] showed a significant improvement in HRQoL (*p* = 0.007; *p* < 0.035; *p* = 0.047).

### Symptom burden

Four studies measured symptom burden [59–61, 65]. Two studies used the Edmonton Symptom Assessment System (ESAS) [61, 65]; Evangelista et al. [65] showed a significant improvement in the total score (*p* < 0.001), while Brannstrom et al. [61] found a significant improvement in nausea in the intervention group (*p* = 0.02). Evangelista et al. [65] showed a significant improvement (*p* < 0.005) in depression measured with the Patient-Health Questionnaire-9 (PHQ-9) as did Delaney et al. (*p* = 0.001) [60].

### Patient activation

Six studies included patient activation or engagement in the intervention description [55, 56, 58, 60–62]. Two studies measured patient activation with the Patient Activation Measure (PAM). Both showed a significant increase in patient activation (*p* < 0.001; *p* = 0.03) [55, 62]. Better symptom recognition and management and additional palliative care support increased patient activation [55] and reduced the uncertainty experienced from high symptom burden, which can undermine patients’ sense of control [51].

### Functional capacity

Ekman et al. [56] found a significant preservation in functional capacity as measured with the Katz ADL (*p* = 0.04). Shively et al. [63] demonstrated a significant improvement in functional capacity with the Medial Outcomes Study Short-Form Health Survey, Veterans adapted version (SF-36V) (*p* = 0.03).

Ekman et al. [56] and Brannstrom et al. [61] showed significant reductions in hospital length of stay (2.5 days shorter, median 6.5, *p* = 0.01) and readmission rates (15 vs. 53, *p* = 0.009), respectively.

## Qualitative data

Three themes were identified from qualitative data where available in the form of participant quotes and related authors’ commentary [52, 53, 56–60, 66]; staff and patient communication; patient engagement; and implementation. Patients appreciated staff empathy [58], trustworthiness, expertise [60] and being listened to by staff [53]. This relationship facilitated patients to become more engaged in their care [53, 60], to negotiate an agreed plan of care [58], to access information [60], to address misconceptions about heart failure [58] and to identify both barriers and available resources to adapt to life with CHF [53, 58, 59].

## Discussion

This is the first review of PCC interventions in CHF. It found that PCC improves HRQoL [60, 61, 65], symptom burden [61, 65], depression [60, 65] and patient activation [55, 61, 62]. Of 10 studies identified, 3 were phase II RCTs and 2 were controlled before and after studies. There are methodological limitations with some studies underpowered due to a small participant number. The strength of evidence is moderate to low; reporting and external validity scored moderately [46]. These findings demonstrate that PCC has a beneficial role in the provision of care to patients with CHF. However, further research is needed to identify the effective components of PCC interventions to inform policy recommendations and clinical practice guidelines.

The interventions had common components including patient assessment, education and healthcare professional–patient collaboration. These commonalities are reflected in the PCC framework where frequently identified domains included healthcare professional–patient collaboration, patient engagement and identification of patient preferences and goals of care. PCC sits within the Innovative Care for Chronic Conditions (ICCC) framework [35] and as a model of care encourages patients’ central role and responsibility for their health care while seeking to address the fragmented healthcare management of these patients with chronic conditions and multi-morbidity experience [36]. Where interventions included patient assessments, these involved a comprehensive assessment of patients’ needs, values and preferences [56, 57, 59–61, 65] which lays the foundation for PCC [37] and better care coordination in chronic disease [36]. Most interventions included education and training to healthcare professionals, patients or both. Training healthcare professionals in patient-centred skills enable them to provide PCC to their patients [42]. Patient education facilitates PCC as well-informed patients are better prepared and ‘activated’ to engage in care discussions [15, 36]. Patient activation describes patients who have the knowledge, skills and motivation to participate and engage in the management of their care [68]. A moderate level of evidence (three RCTs and two controlled before and after studies) demonstrated that interventions which enable patient engagement improve HRQoL [61], symptom burden [61], physical functioning [56, 63] and patient activation [61, 62]. All of the interventions involved multiple patient interactions, which allowed the patient–healthcare professional relationship to develop and is a recognised PCC facilitator [33].

There were common challenges identified across the studies. Recruitment was challenging and 4 studies had ≥20 % attrition rates [56, 58, 59, 66], which is not uncommon in CHF given symptom volatility, high mortality and the subjective nature of the NYHA classification system [69]. Intervention implementation was only partially successful. Qualitative staff interviews by Ekman et al. [56] found that staff given PCC education poorly understood this approach or thought they practiced PCC already [52]. Staff training is dependent on staff ability and willingness to translate received training into clinical practice [70]. PCC interventions designed to involve direct patient contact may be more efficacious than staff training alone [23]. Where interventions involved palliative care or advance care planning, staff felt ill-equipped to have discussions regarding these with patients [57, 66]. This reflects a larger challenge in CHF care where a cultural change is required to increase palliative care awareness and address suboptimal palliative care access [18]. PCC shares a similar philosophy to patient engagement and SDM as palliative care. PCC may prove to be a valuable facilitator to the appropriate integration of palliative care into CHF management, as physical and psychological symptoms are recognised and alleviated in a timely manner and patient activation increased. Embedding a holistic approach to care in usual practice and aligning goals of care to patients’ expressed wishes should encourage consideration of the patient’s management in the context of an illness journey or trajectory rather than in the context of disjointed episodes of decompensation. This should lead healthcare professionals to incorporate a palliative care approach into their own practice or to seek specialist palliative care involvement, where appropriate.

A gap exists between PCC policy recommendations in CHF and clinical practice. No agreement exists as to what PCC should look like in clinical practice for this population. CHF quality indicators include discharge instructions, medication use and smoking cessation [71], but none encompass PCC components. Quality indicators are evidence- or consensus-based measurable markers of practice performance, which can be used to assess the quality of care [72]. This deficit has implications for guideline development and clinical practice. An appraisal of ICD implantation clinical practice guidelines found major deficiencies in decision-making recommendations with an emphasis on device effectiveness and little advice on discussions regarding quality of life or the psychological impact [73]. A British cardiology trainees’ survey supported this finding; only 9.4 % of trainees involved in ICD insertion always discussed the future possibility of device deactivation with patients [74]. Quality indicators identified for patient-centred cancer care include communication, physical support and psychosocial support [75]. NICE in its CHF quality statement identified the following quality measures: personalised patient information; education; support; and the opportunity for patients to increase their understanding of their condition and to be involved in its management [76]. NICE recommend that where no quality indicators exist that quality measures may form a basis for their development [76].

The interventions were multifaceted and complex, and the number of retrieved studies was small. A systematic review of the efficacy of PCC interventions suggests that the challenges associated with designing a complex intervention encompassing this concept may contribute to this paucity of research [8]. However, given that 8 of the 10 included studies were published within the last 5 years, this is a growing body of research. The heterogeneity of outcomes made comparisons difficult and illustrates the challenge in identifying the most appropriate outcome(s) to measure the potential effect of PCC as a multifaceted concept. Five studies identified a primary outcome; improvement in mean symptom burden [61, 65]; patient activation [62]; length of hospital stay (LOS) [56]; and exercise performance [63]. All bar exercise performance showed a significant improvement. Two RCTs demonstrated a significant improvement in their primary outcome; patient activation [62] and nausea, respectively [61]. No study included cost as an outcome measure. PCC reduces readmissions and LOS as shown here and is a strategy to reduce unwanted high-cost interventions by identifying patients’ care preferences [23]. Research is needed into its cost-effectiveness. Few studies included process measures, yet process measures are needed to help identify the effective components of these complex interventions to inform clinical practice.

PCC seeks to improve quality of care by improving patient experience which is of increasing interest at a policy level [19]. Three studies included qualitative research methods to explore the patients’ experience, which gave valuable insights into the potential mechanism of action and effective components of the interventions [52, 56, 58, 59]. The use of qualitative research methods in combination with quantitative research methods helps to answer questions about patient experience which quantitative research methods alone are unable to answer in these complex interventions [77].

## Strengths and limitations

PCC has been a MeSH heading since 1995. Interventions with components of PCC do not necessarily include PCC as a keyword, in the title or abstract. The search strategy was broad to address this and was combined with reference hand searching which retrieved a large number of references. Despite these measures, relevant studies may have been missed. In some papers, intervention components were poorly described resulting in the exclusion of those particular studies. Heart failure disease management clinics are now standard care in CHF with extensive literature on these. Disease management programmes may encompass domains of PCC, but these interventions are frequently poorly described in the literature [78], which presents a challenge when trying to capture all the relevant studies. Bias may have been introduced as the second reviewer only screened 10 % of the titles and abstracts. Screening of all references was undertaken twice by the first reviewer, but given the large number of citations, a relevant paper may still have been missed. The second reviewer was not involved in data extraction. End-of-life terms and non-English studies were excluded, and publication bias could not be formally tested due to the small number of included studies.

## Conclusion

This systematic review has shown that while the strength of evidence for PCC is moderate to poor, there is a small but growing body of evidence which demonstrates that this approach to care reduces symptom burden, readmissions and improves patient activation and quality of life for patients with CHF. Interventions commonly included patient assessment, healthcare professional–patient collaboration, education and patient engagement. Patients’ expertise in their own illness experience was acknowledged [79] as an equal role in the healthcare professional–patient relationship [37]. More research is needed, and future studies should include process measures and quality indicators to help identify the effective components of PCC to inform how policy recommendations can be translated into clinical practice.

## Electronic supplementary material

Supplementary material 1 (PDF 70 kb)
