# Supplementary material for: The gap between policy and practice: a systematic review of patient-centred care interventions in chronic heart failure
Source: Heart Fail Rev. 2015 Oct 5;20(6):673–87. doi: 10.1007/s10741-015-9508-5 (PMC4608978; doi:10.1007/s10741-015-9508-5)
Supplement: Supplementary file 1 — Supplementary material 1 (PDF 70 kb) [file 10741_2015_9508_MOESM1_ESM.pdf]

Title: The gap between policy and practice: a systematic review of patient-centred care interventions in chronic heart failure

Journal: Heart Failure Reviews

Author names: Kane PM, Murtagh FEM, Ryan K, Mahon NG, McAdam B, McQuillan R, Ellis-Smith C, Tracey C, Howley C, Raleigh C, O'Gara G, Higginson IJ, Daveson BA, on behalf of BuildCARE

Corresponding author
Dr Pauline M Kane, Cicely Saunders International PhD Clinical Research Fellow, King's College London, Cicely Saunders Institute, Department of Palliative Care, Policy and Rehabilitation, Bessemer Road, London SE5 9PJ, UK.

E-mail: pauline.m.kane@kcl.ac.uk

Search strategy used for Medline

1. exp self care/
2. ("self care" or self-care).mp.
3. self-manage*.mp.
4. 1 or 2 or 3
5. exp Patient-Centered Care/
6. patient-centered.mp.
7. patient-centred.mp.
8. person-centered.mp.
9. person-centred.mp.
10. patient-orientated.mp.
11. person-orientated.mp.
12. patient-focused.mp.
13. person-focused.mp.
14. exp professional-patient relations/
15. professional-patient relations.mp.
16. 14 or 15
17. exp professional-family relations/
18. professional-family relations.mp.
19. 17 or 18
20. exp patient participation/
21. exp Patient Care Planning/
22. exp education/
23. education.mp.
24. 22 or 23

25. (16 or 19) and 24
26. 5 or 6 or 7 or 8 or 9 or 10 or 11 or 12 or 13 or 20 or 21 or 25
27. decision making.mp.
28. exp choice behavior/
29. (share* adj decision adj making).mp.
30. (decision adj analys*).mp.
31. 27 or 28 or 29 or 30
32. (patient or subject or person).mp.
33. (family or carer or caregiver).mp.
34. (professional or physician or clinician or practitioner).mp.
35. (32 and 34) or (33 and 34)
36. professional-patient relations.mp.
37. physician-patient relations.mp.
38. 35 or 36 or 37
39. (shar* adj information).mp.
40. (patient adj choice*).mp.
41. (patient adj understanding).mp.
42. ((check or clarify) adj3 understanding).mp.
43. physician preferences.mp.
44. (treatment adj options).mp.
45. values.mp.
46. preferen*.mp.
47. (communicat* adj risk).mp.
48. attitude of health personnel.mp.
49. (patient adj expect*).mp.
50. (problem adj definite*).mp.
51. (ask adj question*).mp.
52. (assess adj risk).mp.
53. self-manag*.mp.
54. (self-care or "self care").mp.
55. (decision adj aids).mp.
56. decision support techniques.mp.
57. checklist.mp.
58. 55 or 56 or 57
59. (goal adj set*).mp.
60. negotiat*.mp.
61. deliberat*.mp.
62. (decis* and mak*).mp.
63. consensus.mp.
64. concordance.mp.
65. agreement.mp.
66. (action adj plan).mp.
67. or/59-66
68. (treatment adj (compliance or concordance)).mp.
69. 39 or 40 or 41 or 42 or 43 or 44 or 45 or 46 or 47 or 48 or 49 or 50 or 51 or 52 or 53 or 54
70. (31 and 38) or (38 and 69) or (38 and 58) or (38 and 67) or (31 and 68) or (69 and 68)
71. patient empowerment.mp.
72. 4 or 26 or 70 or 71
73. exp heart failure/
74. heart failure.mp.

75. chronic heart failure.mp.
76. congestive cardiac failure.mp.
77. congestive heart failure.mp.
78. (advanced adj6 heart failure).mp.
79. exp congestive heart failure/
80. dilated cardiomypathy.mp.
81. hf.ti,ab.
82. chf.ti,ab.
83. ccf.ti,ab.
84. cardiac failure.mp.
85. 73 or 74 or 75 or 76 or 77 or 78 or 79 or 80 or 81 or 82 or 83 or 84
86. exp "Quality of Life"/
87. quality of life.mp.
88. QOL.mp.
89. QL.mp.
90. HRQOL.mp.
91. HRQL.mp.
92. life quality.mp.
93. well being.mp.
94. well-being.mp.
95. satisfaction.mp.
96. symptom relief.mp.
97. symptom$.mp.
98. health perception*.mp.
99. patient satisfaction*.mp.
100. "health-related quality of life".mp.
101. 86 or 87 or 88 or 89 or 90 or 91 or 92 or 93 or 94 or 95 or 96 or 97 or 98 or 99 or 100
102. (anxious* or anxiet* or agitat* or restless* or panic* or stress* or nervous*).mp.
103. exp anxiety/
104. 102 or 103
105. exp depression/
106. depression.mp.
107. depress*.mp.
108. Pain/
109. pain.mp.
110. Dyspnea/
111. (shortness adj2 of adj2 breath?).mp.
112. breathlessness.mp.
113. dyspn?ea.mp.
114. Edema/
115. ?edema.mp.
116. (fluid adj3 retention).mp.
117. (swelling adj6 heart).mp.
118. Fatigue/
119. fatigue.mp.
120. tired*.mp.
121. ((poor or low or reduced or less or no) adj3 energy).mp.
122. cough/
123. cough.mp.

124. 104 or 105 or 106 or 107 or 108 or 109 or 110 or 111 or 112 or 113 or 114 or 115 or 116 or 117 or 118 or 119 or 120 or 121 or 122 or 123
125. Communication/
126. communicat*.mp.
127. 125 or 126
128. 101 or 124 or 127
129. 85 and 72 and 128

Table 5 Excluded studies

Study	Exclusion
**Agrinier 2013[1]**	Disease Management Programme, no shared decision making
**Atienza 2004[2]**	Disease Management Programme, no shared decision making
**Bocchi 2008[3]**	Disease Management Programme, no shared decision making
**Butler 2015[4]**	Non-interventional study
**Chang 2014[5]**	Review: not primary research
**Ciccone 2010[6]**	CHF data not reported separately
**Cohen 2014[7]**	Abstract available only, conference proceedings
**Collins 2013 [8]**	Review: not primary research
**Creber 2014[9]**	Abstract available only, conference proceedings
**Cruz 2010[10]**	Disease Management Programme, no shared decision making
**Di Salvo 2003[11]**	Disease Management Programme, no shared decision making
**Doughty 2002[12]**	Disease Management Programme, no shared decision making
**Doris Sau-Fung 2014[13]**	Abstract available only, conference proceedings
**Frazee2012[14]**	Abstract available only, conference proceedings
**Goge 2010[15]**	Abstract available only, conference proceedings
**Grady 2014[16]**	Education intervention, no shared decision making
**Harrison 2007[17]**	Education intervention, no shared decision making
**Hadjistavropoulos 2008[18]**	Non-interventional study
**Hershberger 2005[19]**	Disease Management Programme, no shared decision making
**Hershberger 2001[20]**	Disease Management Programme, no shared decision making
**Hinterbuchner 2010[21]**	Abstract available only, conference proceedings
**Houston-Feenstra 2010[22]**	Education intervention, no shared decision making
**Kodiath 2005[23]**	Self-care intervention, no shared decision making
**Kwa 2013[24]**	Abstract available only, conference proceedings
**LaFramboise 2003[25]**	Telemedicine intervention only
**Larina 2012[26]**	Abstract available only, conference proceedings
**Leventhal 2011[27]**	Disease Management Programme, no shared decision making
**Lin 2008[28]**	Disease Management Programme, no shared decision making
**Lorig 1999[29]**	Disease Management Programme, no shared decision making
**Otsu 2012[30]**	Disease Management Programme, no shared decision making
**Patel 2008[31]**	Disease Management Programme, no shared decision making
**Piepoli 2006[32]**	Disease Management Programme, no shared decision making
**Rich 1995[33]**	Disease Management Programme, no shared decision making
**Record 2010[34]**	Abstract available only, conference proceedings
**Ryder 2011[35]**	Review: not primary research
**Schwarz 2011[36]**	Review: not primary research
**Schwarz 2011[37]**	Review: not primary research
**Sethers 2012[38]**	Abstract available only, conference proceedings
**Smith 2014[39]**	Abstract available only, conference proceedings
**Stewart 2012[40]**	Disease Management Programme, no shared decision making
**Tong 2013[41]**	Abstract available only, conference proceedings
**Waterworth 2010[42]**	Review: not primary research
**Ylonen 2012[43]**	Education intervention, no shared decision making

References

1. Agrinier N, Altieri C, Alla F, Jay N, Dobre D, Thilly N, et al. (2013) Effectiveness of a multidimensional home nurse led heart failure disease management program - A French nationwide time-series comparison. Int J Cardiol. 168:3652–8.
2. Atienza F, Anguita M, Martinez-Alzamora N, Osca J, Ojeda S, Almenar L, et al. (2004) Multicenter randomized trial of a comprehensive hospital discharge and outpatient heart failure management program. Eur J Heart Fail. 6:643–52.
3. Bocchi EA, Cruz F, Guimaraes G, Pinho,Moreira,L.F., Issa VS, Ayub,Ferreira,S.M., et al. (2008) Long-term prospective, randomized, controlled study using repetitive education at six-month intervals and monitoring for adherence in heart failure outpatients: the REMADHE trial. Circ Heart Fail. 1:115–24.
4. Butler J, Binney Z, Kalogeropoulos A, Owen M, Clevenger C, Gunter D, et al. (2015) Advance directives among hospitalized patients with heart failure. JACC: Heart Failure. 3:112–21.
5. Chang S, Newton PJ, Inglis S, Luckett T, Krum H, Macdonald P, et al. (2014) Are all outcomes in chronic heart failure rated equally? An argument for a patient-centred approach to outcome assessment. Heart Fail Rev. 19:153–62.
6. Ciccone MM, Aquilino A, Cortese F, Scicchitano P, Sassara M, Mola E, et al. (2010) Feasibility and effectiveness of a disease and care management model in the primary health care system for patients with heart failure and diabetes (Project Leonardo). Vasc Health Risk Manag. 6:297–305.
7. Cohen B, Cohen JL, Stuart-Shor EM. (2014) Integration of an advanced heart failure pathway in a rural community-based multispecialty practice. Circulation: Conference American Heart Association's 2014 Scientific Sessions and Resuscitation Science Symposium Chicago, IL United States.
8. Collins SP, Storrow AB. (2013) Moving toward comprehensive acute heart failure risk assessment in the emergency department: The importance of self-care and shared decision making. JACC: Heart Failure. 1:273–80.
9. Creber R, Patey M, Riegel B. (2014) Motivational interviewing tailored intervention to promote heart failure self-care (MITI-HF): Primary study results. Circulation.;Conference American Heart Association's 2014 Scientific Sessions and Resuscitation Science Symposium Chicago, IL United States. Conference Start 20141115 Conference End 20141118. Conference Publication.
10. Cruz F, Issa VS, Ayub-Ferreira SM, Chizzola PR, Souza G, Moreira L, et al. (2010) Effect of a sequential education and monitoring programme on quality-of-life components in heart failure. Euro J Heart Fail. 12:1009–15.
11. Di,Salvo,T.G., Warner,Stevenson,L. (2003) Interdisciplinary team-based management of heart failure. Dis Manag Health Outcomes. 11:87–94.
12. Doughty RN, Wright SP, Pearl A, Walsh HJ, Muncaster S, Whalley GA, et al. (2002) Randomized, controlled trial of integrated heart failure management: The Auckland heart failure management study. Eur Heart J. 23:139–46.
13. Doris Sau-Fung Yu DS-F, Lee DT, Stewart S, Thompson, Choi KC, Yu CM. (2014) The effects of a nurse-led empowerment-based disease management program on clinical outcomes, self-care and health-related quality of life among Chinese patients with heart failure. Eur J Heart Fail. 16:60.
14. Frazee S, Schenck J. (2012) Implement goal of the day to improve patient and family centered care. Heart and Lung: Journal of Acute and Critical Care.;Conference 8th Annual Conference of the American Association of Heart Failure Nurses, AAHFN 2012 Chicago, IL United States. Conference Start 20120628 Conference End 20120630. Conference Publication:426.
15. Goge S, Sherrard H, Kearns S, MacPhee E, Struthers C, Frattini E. (2010) Implementation of the heart failure discharge tool. Can J Cardiol.;Conference 2010 Canadian Council of Cardiovascular Nurses Annual Scientific Sessions Montreal, QC Canada. Conference Start 20101023 Conference End 20101026. Conference Publication:155D-156D.
16. Grady KL, de,Leon,C.F., Kozak AT, Cursio JF, Richardson D, Avery E, et al. (2014) Does self-management counseling in patients with heart failure improve quality of life? Findings from the Heart Failure Adherence and Retention Trial (HART). Qual Life Res. 23:31–8.

17. Harrison MB, Graham ID, Logan J, Toman C, Friederg E. (2007) Evidence to practice: Pre-post-implementation study of a patient/provider resource for self-management with heart failure. Int J Evid Based Healthc. 5(1):92–101.
18. Hadjistavropoulos HD, Dunn-Pierce T, Biem HJ. (2008) Provider perceptions of implementation of integrated care pathways for patients with chronic heart conditions. Can J Cardiovasc Nurs. 18:20–6.
19. Hershberger RE, Nauman DJ, Byrkit J, Gillespie G, Lackides G, Toy W, et al. (2005) Prospective evaluation of an outpatient heart failure disease management program designed for primary care: The Oregon model. J Card Fail. 11:293–8.
20. Hershberger RE, Ni H, Nauman DJ, Burgess D, Toy W, Wise K, et al. (2001) Prospective evaluation of an outpatient heart failure management program. J Card Fail. 7:64–74.
21. Hinterbuchner L, Fritsch MF. (2010) CardioMobile home care for congestive heart failure. Eur J Cardiovasc Nurs.;Conference 10th Annual Spring Meeting on Cardiovascular Nursing Geneva Switzerland. Conference Start 20100312 Conference End 20100313. Conference Publication:S30.
22. Houston-Feenstra L, Chiong JR, Pina I. (2010) Outcomes of a medical home model for the management of COPD and heart failure. Chest;Conference CHEST 2010 Annual Meeting Vancouver, BC Canada. Conference Start 20101030 Conference End 20101104. Conference Publication.
23. Kodiath M, Kelly A, Shively M. (2005) Improving quality of life in patients with heart failure: an innovative behavioral intervention. J Cardiovasc Nurs. 20:43–8.
24. Kwa W, Chong R, Wong J, Koh A, Goh CY, Lee HH, et al. (2013) Advance care planning is beneficial in chronic heart failure patients-a pilot study. Heart.;Conference Annual Scientific Sessions of the Asia Pacific Heart Association 2013 Singapore Singapore. Conference Start 20130419 Conference End 20130421. Conference Publication:A58.
25. LaFramboise LM, Todero CM, Zimmerman L, Agrawal S. (2003) Comparison of HEALTH BUDDY with traditional approaches to heart failure management. Fam Community Health.;26:275–88.
26. Larina V, Bart B, Mikhaylusova M, Golovko M. (2012) Effects of person-centred care in outpatients above 60 years with chronic heart failure. Eur J Prev Cardiol.;Conference EuroPRevent 2012 Dublin Ireland. Conference Start 20120503 Conference End 20120505. Conference Publication:S113.
27. Leventhal ME, Denhaerynck K, Brunner-La RHP, Burnand B, Conca-Zeller A, Bernasconi AT, et al. (2011) Swiss Interdisciplinary Management Programme for Heart Failure (SWIM-HF): a randomised controlled trial study of an outpatient inter-professional management programme for heart failure patients in Switzerland. Swiss Medical Wkly. 141:w13171.
28. Lin A, Cavendish J, Boren D, Ofstad T, Seidensticker D. (2008) A pilot study: reports of benefits from a 6-month, multidisciplinary, shared medical appointment approach for heart failure patients. Mil Med. 173:1210–3.
29. Lorig KR, Sobel DS, Stewart AL, Brown, Byron William Jr., Bandura A, Ritter P, et al. (1999) Evidence suggesting that a chronic disease self-management program can improve health status while reducing hospitalization. Med Care. 37:5–14.
30. Otsu H, Moriyama M. (2012) Follow-up study for a disease management program for chronic heart failure 24 months after program commencement. Jpn J Nurs Sci. 9:136–48.
31. Patel H, Shafazand M, Ekman I, Hojgard S, Swedberg K, Schaufelberger M. (2008) Home care as an option in worsening chronic heart failure- A pilot study to evaluate feasibility, quality adjusted life years and cost-effectiveness. Eur J Heart Fail. 10:675–81.
32. Piepoli MF, Villani GQ, Aschieri D, Bennati S, Groppi F, Pisati MS, et al. (2006) Multidisciplinary and multisetting team management programme in heart failure patients affects hospitalisation and costing. Int J Cardiol. 111:377–85.
33. Rich MW, Beckham V, Wittenberg C, Leven CL, Freedland KE, Carney RM. (1995) A multidisciplinary intervention to prevent the readmission of elderly patients with congestive heart failure. N Engl J Med. 333:1190–5.
34. Record J, Rand C, Hanyok L, Federowicz M, Hellmann D, Ziegelstein R. (2010) A hospital-based curriculum for patient-centered care and safe transitions: Effect on 30-day heart failure

readmissions. J Hosp Med. Conference 2010 Annual Meeting of the Society of Hospital Medicine, SHM 2010 Washington, DC United States. Conference Start 20100408 Conference End 20100411. Conference Publication:65–6.

35. Ryder M, Beattie JM, O'Hanlon R, McDonald K. (2011) Multidisciplinary heart failure management and end of life care. Curr Opin Support Palliat Care. 5:317–21.
36. Schwarz ER, Cleenewerck L, Phan A, Bharadwaj P, Hobbs R. (2011) Philosophical implications of the systemic and patient-oriented management of chronic heart failure. J Rel Health. 50:348–58.
37. Schwarz ER, Philip KJ, Simsir SA, Czer L, Trento A, Finder SG, et al. (2011) Maximal care considerations when treating patients with end-stage heart failure: Ethical and procedural quandaries in management of the very sick. J Rel Health. 50:872–9.
38. Sethares KA, Asselin ME (2012) Feasibility of a brief guided reflective intervention to improve self-care in patients recently hospitalized with heart failure. Heart Lung. Conference 8th Annual Conference of the American Association of Heart Failure Nurses, AAHFN 2012 Chicago, IL United States. Conference Start 20120628 Conference End 20120630. Conference Publication:417–8.
39. Smith HE. (2014) A novel palliative care program for patients with chronic heart failure that decreased readmission rates. J Card Fail. Conference 18th Annual Scientific Meeting of the Heart Failure Society of America, HFSA 2014 Las Vegas, NV United States. Conference Start 20140914 Conference End 20140917. Conference Publication:S59.
40. Stewart S, Carrington MJ, Marwick TH, Davidson PM, Macdonald P, Horowitz JD, et al. (2012) Impact of home versus clinic-based management of chronic heart failure: The WHICH? (Which heart failure intervention is most cost-effective & consumer friendly in reducing hospital care) multicenter, randomized trial. J Am Coll Cardiol. 60:1239–48.
41. Tong K, Myers S, Poole P, Nguyen J, Griffin E, Levich B. (2013) A multidisciplinary approach at the primary care level improves heart failure care. J Card Fail. Conference 17th Annual Scientific Meeting Heart Failure Society of America, HFSA 2013 Orlando, FL United States. Conference Start 20130922 Conference End 20130925. Conference Publication:S42-S43.
42. Waterworth S, Gott M. (2010) Decision making among older people with advanced heart failure as they transition to dependency and death. Curr Opin Support Palliat Care.;4:238–42.
43. Ylonen K, Heikkila J. (2012) HF patient education: Patient-or nurse-centered. Eur J Heart Fail, Supplement.;Conference Heart Failure 2012 Belgrade Serbia. Conference Start 20120519 Conference End 20120522. Conference Publication:S163.
